# Effect of Strength Enhancement of Soil Treated with Environment-Friendly Calcium Carbonate Powder

**DOI:** 10.1155/2014/526491

**Published:** 2014-02-04

**Authors:** Kyungho Park, Sangju Jun, Daehyeon Kim

**Affiliations:** Department of Civil Engineering, Chosun University, Gwangju 501-759, Republic of Korea

## Abstract

This study aims to investigate the effects of the strength improvement of soft ground (sand) by producing calcium carbonate powder through microbial reactions. To analyze the cementation effect of calcium carbonate produced through microbial reaction for different weight ratios, four different types of specimens (untreated, calcium carbonate, cement, and calcium carbonate + cement) with different weight ratios (2%, 4%, 6%, and 8%) were produced and cured for a period of 3 days, 7 days, 14 days, 21 days, and 28 days to test them. The uniaxial compression strength of specimens was measured, and the components in the specimen depending on the curing period were analyzed by means of XRD analysis. The result revealed that higher weight ratios and longer curing period contributed to increased strength of calcium carbonate, cement, and calcium carbonate + cement specimens. The calcium carbonate and the calcium carbonate + cement specimens in the same condition showed the tendency of decreased strength approximately 3 times and two times in comparison with the 8% cement specimens cured for 28 days, but the tendency of increased strength was approximately 4 times and 6 times in comparison with the untreated specimen.

## 1. Introduction 

### 1.1. Background and Objective

Recent increasing cost of raw materials, scarcity of natural materials, and lack of construction materials result in difficulty in developing the construction industry. Fast industrial development contributes to qualitative and quantitative expansion of the national key industry, so Korea experiences difficulty in securing sites in comparison with other countries with large land areas. This leads to Korea's interest in methods of improving soft ground of loose sandy soil or weak silt which has not been considered as a construction site, for efficient use of land. Many construction companies have taken a lot of overseas orders from Middle East countries to work there, and it is thus needed to develop a new method of or new material for improving soft ground.

The Korea Cement Association (2005) says that the volume of cement produced in Korea in 2006 amounts to approximately 48 million tons which is the 7th place in the global volume of produced cement, and carbon dioxide discharged in producing 1 ton of cement is approximately 0.9 tons. Because carbon dioxide discharged through cement is a major cause of greenhouse gas, various studies are currently underway to develop substitutes for reducing cement to address the environmental issue.

In this study, the effect of the strength improvement of soft ground was analyzed when calcium carbonate produced by microbes was applied to loose sandy soil which is weak ground. To this end, the calcium carbonate was produced as powder like cement to compare it with cement. For analyzing and comparing their applicability with cement, calcium carbonate + cement specimens were produced to analyze their strength through the uniaxial compression test after curing them for 3 days, 7 days, 14 days, 21 days, and 28 days. The XRD analysis was carried out in order to examine chemical reaction and to analyze components of each specimen depending on longer curing period.

### 1.2. Previous Studies

Various substitutes have been studied for reducing consumed cement. Some Korean and overseas researchers have studied how to use cement created through biological and chemical reactions of *B. pasteurii* (KCTC 3558) which is one type of numerous microbes under the ground to cement soft ground Kim et al., [1], [2–8], K. H. Park et al., [9], K. H. Park and D. H. Park [3, 10].

Kim et al. [1] are the first Korean researchers who have studied how to use microbes in order to enhance the strength of soft ground. They developed a method of increasing precipitation of cement by means of the microbial condition of high concentration. They now own a patent of microbial cementation registered in the Korean Intellectual Patent Office and titled “Method of Cementing Soft Ground by Using Microbes” [11]. Microbial cementation of soft ground was identified through a test of mixing an untreated specimen, a high concentration-treated specimen, and a normal concentration-treated specimen [12].

Park et al. [2] intended to cement the ground by using a plant extract with a component of urease to precipitate calcium carbonate. They found that the strength of the specimen using the plant extract was increased to about 300 kPa, approximately 3 times higher than that of the untreated specimen. However, the process of getting the primary and the secondary liquid from the plant extract is not simple, and maximum 5% of cement is obtained after 4 days. Therefore, it takes several days to have the maximum amount of cement even after mixing the extract with the reaction solution.

In other countries, some researchers including Paassen et al. [7] (2008) and DeJong et al. [5, 6] have studied microbial cementation. DeJong et al. [5] achieved a desired strength through triaxial compression strength by continuous injection, but the process is very complicated. Whiffin et al. [8] obtained a great value of 10 MPa for the specimen strength but estimated the strength with presumed values, not through quantitative analysis. Paassen et al. (2008) carried out a test in a model soil tank. The test results revealed that the strength increased along the surface and the part where air is available enough, but slight strength increase was observed in the central part which did not have enough air.

Paassen et al. [7] (2008), DeJong et al. [5, 6], Whiffin et al. [8], and Park et al. [2] used an aqueous solution of calcium carbonate to enhance the strength of soft ground. However, calculation of strength by means of estimates is not highly reliable, and the aqueous solution of calcium carbonate lowers strength, and it is not easy to handle the supernatant liquid if much of the solution is injected. The calcium carbonate used in this study is produced through the reaction between a microbial solution and an aqueous solution of calcium chloride. It is produced as powder like cement which is widely used as a construction material, and it is possible to identify the effect of increased quantitative strength, unlike prior studies.

## 2. Method of Producing Specimens

### 2.1. Materials Used in the Test

#### 2.1.1. Sand and Cement

**Figure 1:**
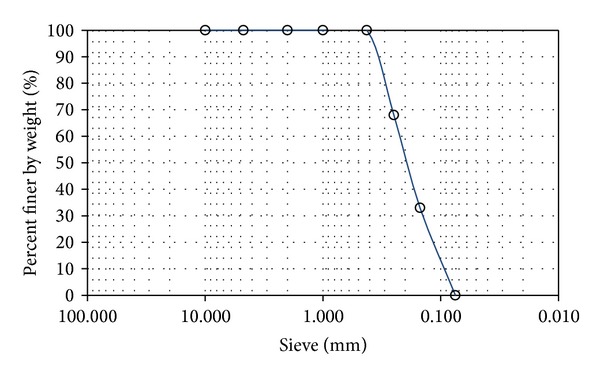
Particle size of the sample test results.

The sample sand was collected from the riverside of the Seomjingang River, Sangdong-myon, Namwon, Jeollabuk-do. Sieve analysis was performed for the collected sample to use the sample mixed in the ratio of 1 : 1 : 1 with the sample remaining in the sieves #60 (0.25 mm), #100 (0.15 mm), and #200 (0.075 mm) in order to produce self-standing specimens to measure strength through the uniaxial compression test.  Figure 1 shows the result of soil analysis for the sand sample used in this study. Here, the result of calculation of the coefficient of uniformity and the curvature with the particle-size distribution curve is 2.63 for the coefficient of uniformity (*C*_*u*_) and 0.94 for the coefficient of curvature (*C*_*g*_), which means SP sample of nonuniform particle size. The cement was Portland cement from a Korean company.

#### 2.1.2. Calcium Carbonate Produced through Microbial Reaction

*B. pasteurii* (KCTC 3558) used in this study was purchased for bacterial culture from the Korean Collection for Type Culture of Korea Research Institute of Bioscience and Biotechnology. The culture medium for culturing *B. pasteurii *was Nutrient Broth 8 g/L, Urea 20 g/L. It was mixed with 1 liter of distilled water, and *B. pasteurii* was cultured in a shaking incubator of 180 RPM at 30°C. Equation (1) is for the reaction between microbes and urea. Growing microbes react with urea to be hydrolyzed into a carbonate ion (CO_3_^2−^) and two ammonium ions (NH_4_^+^):

(1) $$\begin{array}{c} \text{CO}{\left(\text{N}{\text{H}}_{2}\right)}_{2}+2{\text{H}}_{2}\text{O}\overset{\text{Urease}\;\text{reaction}}{\to }{{\text{CO}}_{3}}^{2-}+2{{\text{NH}}_{4}}^{+}. \end{array}$$

Calcium carbonate (CaCO_3_) is produced by the reaction between carbonate ions (CO_3_^2−^) produced through which *B. pasteurii* takes urea and the calcium ions (Ca^2+^) in the aqueous solution of calcium chloride (CaCl_2_). Equation (2) is for the precipitation reaction of calcium carbonate:

(2) $$\begin{array}{c} {{\text{CO}}_{3}}^{2-}+\text{C}{\text{a}}^{2+}⟶\text{CaC}{\text{O}}_{3}. \end{array}$$

**Figure 2:**
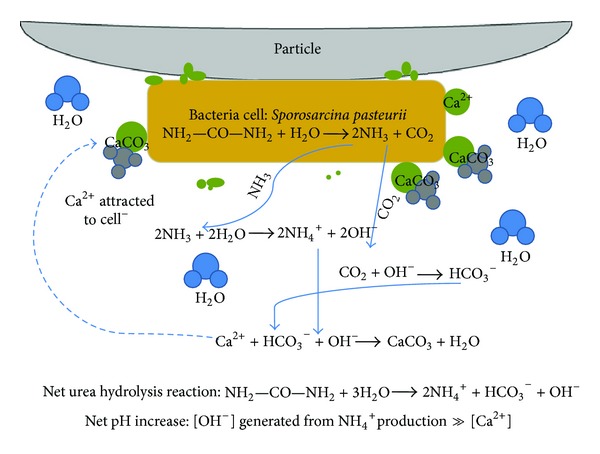
Process of producing calcium carbonate [6].

The calcium carbonate (CaCO_3_) is produced through microbial reaction in the process described above.  Figure 2 shows the process of producing calcium carbonate and the cycle of precipitating soil particles in each equation.

#### 2.1.3. Powdering Calcium Carbonate

**Figure 3:**
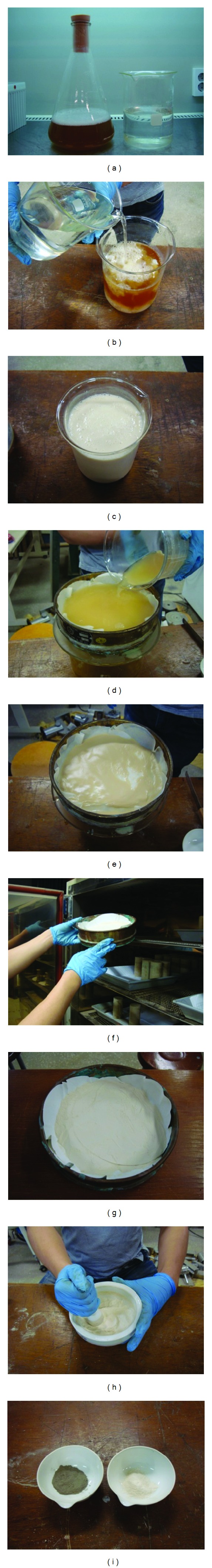
Extracting and producing calcium carbonate.

The calcium carbonate used in this study was produced through microbial reaction and then powdered like cement, unlike prior studies for which an aqueous solution of calcium carbonate was used. The microbial reaction was done by adding calcium chloride into bacteria solution. The powdered calcium carbonate enabled the supernatant liquid to be handled and quantitative strength to be measured, which was an issue in prior studies.  Figure 3 shows the process of extracting, drying, and powdering calcium carbonate from the microbial solution and the aqueous solution of calcium chloride. In the method described below, the calcium carbonate produced through microbial reaction was used as a cementation agent.

Figure 3 shows (a) a medium for microbes (1L); (b) mixing the aqueous solution of calcium chloride (1L); (c) precipitating calcium carbonate in the reaction solution; (d) filtering the reaction solution; (e) extracting calcium carbonate; (f) drying calcium carbonate at 40°C for 24 hours; (h) powdering calcium carbonate.

### 2.2. Producing Specimens

**Figure 4:**
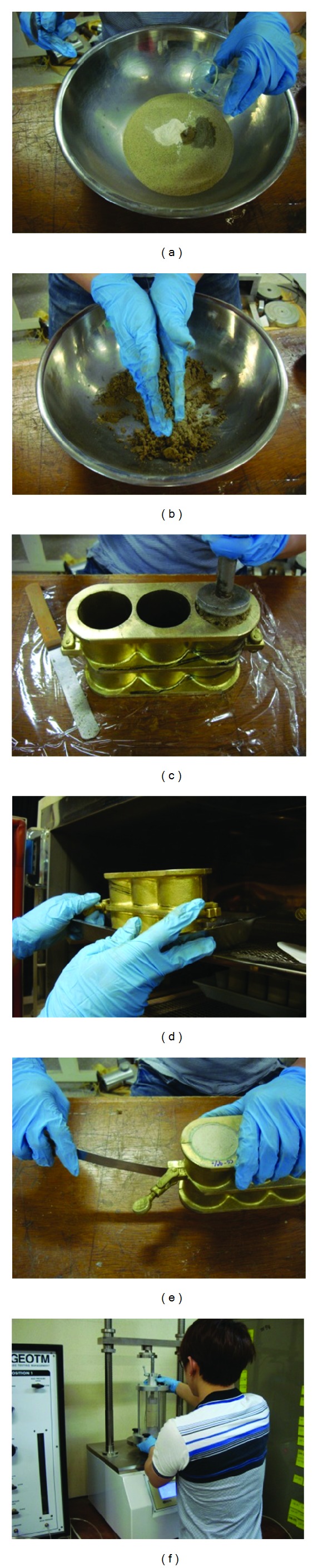
Mixing sample materials and producing specimens.

Specimens of which the ratio of height to the diameter were produced to be 1 : 2 in the ratio of the diameter (D) 5 cm to the height (H) 10 cm as shown in Figure 4 in conformity with the Korea Industrial Standard KS F2314. This aimed to measure the uniaxial compression strength of the specimens of calcium carbonate (produced through microbial reaction), cement, and calcium carbonate + cement. The process applied was (a) to measure the weight of the sample material, the cement, and the water, respectively, (b) to hand-mix the sample material, (c) to produce specimens in a mold through 3-layer compaction, (d) to cure the specimens at 40°C, (e) to remove the cured specimen mold, and (f) to measure the uniaxial compression strength of the specimens.

The 65 produced specimens were divided into 4 cases of 5 untreated specimens, 20 calcium carbonate specimens, 20 cement specimens, and 20 calcium carbonate + cement specimens. The untreated specimens were produced to correspond to 3 days, 7 days, 14 days, 21 days, and 28 days. Five specimens for each of calcium carbonate, cement, and calcium carbonate + cement were produced to have the weight ratios of 2%, 4%, 6%, and 8% to measure the strength depending on the curing period (3 days, 7 days, 14 days, 21 days, and 28 days).  Table 1 shows the mixing ratio of the specimens untreated, calcium carbonate, cement, and calcium carbonate + cement, with which 5 specimens were produced, respectively.

## 3. Analysis of Test Results

### 3.1. Uniaxial Compression Test Result of Untreated Specimens

**Figure 5:**
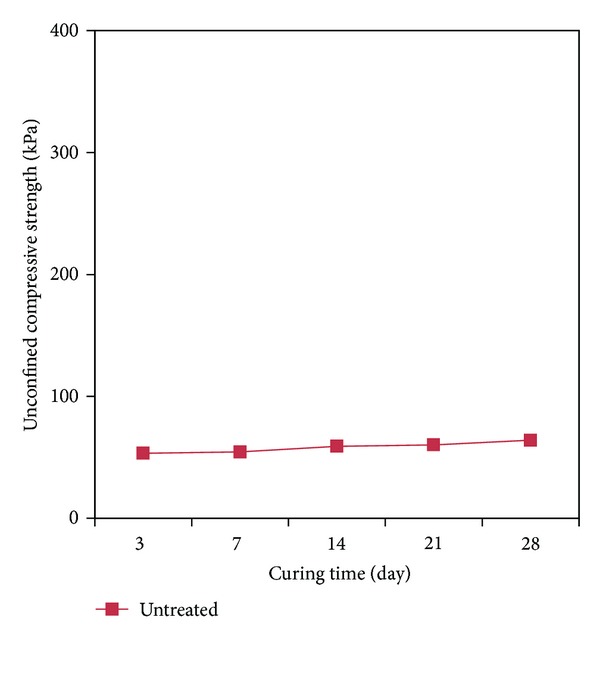
Curing period to compare strength of untreated specimens.

The uniaxial compression strength test was carried out for the specimens at a shearing speed of 1%/min.  Table 2 and Figure 5 show the results of uniaxial compression strength test for the untreated specimens after curing them for 3 days, 7 days, 14 days, 21 days, and 28 days.

The untreated sand specimens to which the cementation agent was not added stood for themselves as a circular cylinder by means of apparent cohesion, and the uniaxial compression strength thereof was 53.42 kPa for the initial curing (3 days), which is shown in Table 2. For curing for 28 days, the result was 64.10 kPa, which increased approximately 1.2 times. This increase is due to the drying moisture in the specimens.

### 3.2. Results of Uniaxial Compression Test for Calcium Carbonate Specimens

**Figure 6:**
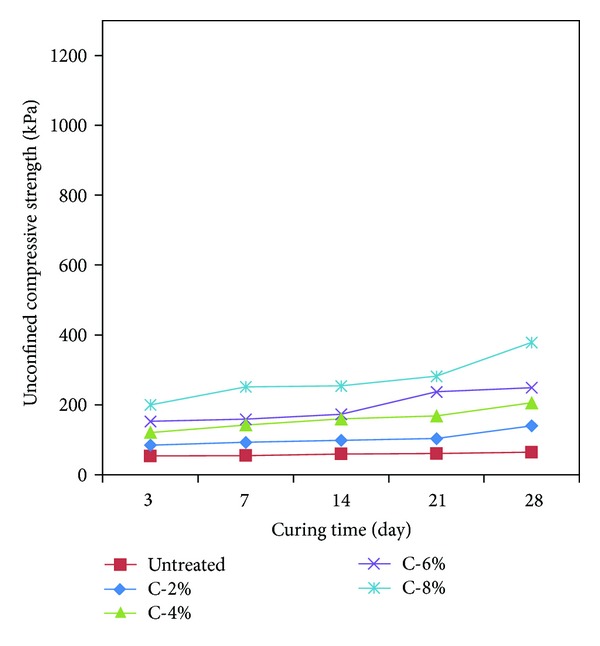
Curing period to compare the strength of calcium carbonate specimens.

The calcium carbonate specimens were tested at the shearing speed of 1%/min in the same method as the uniaxial compression strength test for untreated specimens. The result of the uniaxial compression test for the calcium carbonate specimens after curing them for 3 days, 7 days, 14 days, 21 days, and 28 days is shown in Table 3 and Figure 6. As shown in Table 3, the initial strength (3 days) of the calcium carbonate specimens was 84.57–199.95 kPa, which exhibits increased strength approximately 2.4 times with the increased weight ratio. It is thought that the increased amount of calcium carbonate affects the increased strength of the sand ground. The strength increased approximately 2.6-2.7 times with the increased weight ratio for the curing period of 7 days, 14 days, 21 days, and 28 days. The calcium carbonate specimens also exhibited increased strength with the longer curing period like the untreated specimens.

**Figure 7:**
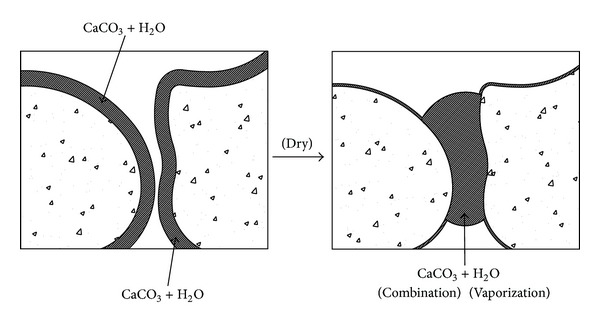
Effect of enhancing strength of calcium carbonate specimens.

As shown in Figure 7, it is thought that the uniaxial compression strength of the calcium carbonate specimens increased with the longer curing period. While the calcium carbonate powder was mixed with water and then dried around the sand particles in producing the specimens, evaporating water contributed to shrinking for holding sand particles. The strength of the calcium carbonate specimens increased approximately minimum 1.6 times for the curing period of 3 days in comparison with the untreated specimens.

### 3.3. Results of Uniaxial Compression Test of Cement Specimens

**Figure 8:**
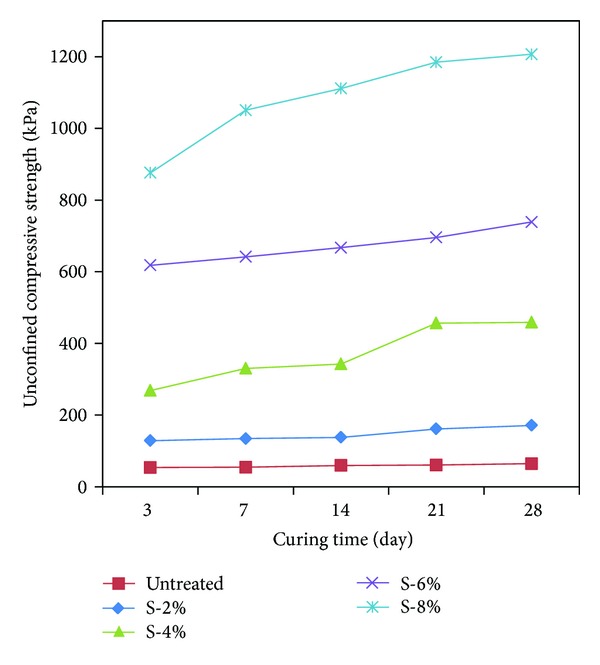
Curing period to compare the strength of cement specimens.

The uniaxial compression test was carried out for the cement specimens in the same method as the one for the untreated and the calcium carbonate specimens described above.  Table 4 and Figure 8 show the result of the uniaxial compression strength test for the cement specimens after curing them for the period of 3 days, 7 days, 14 days, 21 days, and 28 days. The initial strength (3 days) of the cement specimens was 128.45–876.06 kPa, which increased approximately 6.8 times with the increased ratio, even higher than the strength of the untreated and the calcium carbonate specimens previously tested. This proves the effect of enhanced strength of ordinary cement specimens.

**Figure 9:**
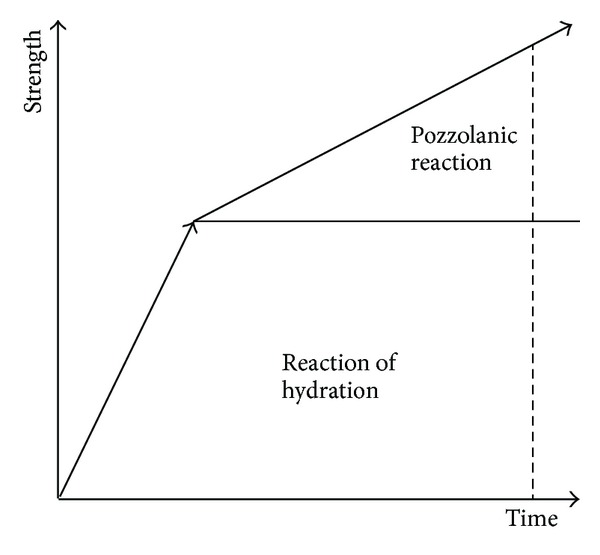
Enhanced strength through ground improvement with cement.

It was seen that the cement specimens also exhibited increased strength approximately 7.0-8.0 times with the increased weight ratio for the curing period of 7 days, 14 days, 21 days, and 28 days. It is thought that the reaction of hydration and the Pozzolanic reaction of cement contributed to the effect of even enhanced strength with longer curing period than the enhanced strength of the untreated and the calcium carbonate specimens previously tested. The effect of enhanced strength is shown in Figure 9. The strength of the cement specimens increased approximately 16.4–18.8 times in comparison with the untreated specimens.

### 3.4. Results of Uniaxial Compression Test of Calcium Carbonate + Cement Specimens

**Figure 10:**
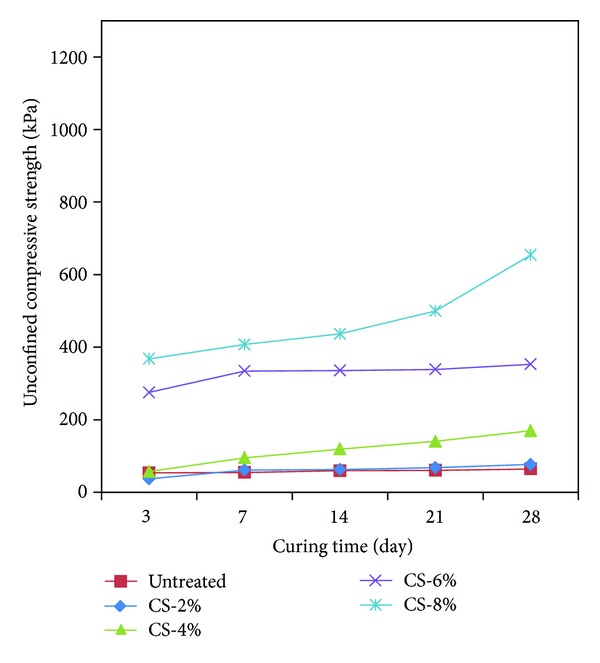
Curing period to compare the strength of calcium carbonate + cement specimens.

The uniaxial compression strength of the calcium carbonate + cement specimens was tested at a shearing speed of 1%/min in the same method as the one for the previous specimens. The uniaxial compression strength test of the calcium carbonate + cement specimens was carried out by mixing calcium carbonate with cement to determine compatibility as an admixture.  Table 5 and Figure 10 show the result of uniaxial compression test for the calcium carbonate + cement specimens after curing them for 3 days, 7 days, 14 days, 21 days, and 28 days. The initial strength (3 days) of the calcium carbonate + cement specimens was 36.80–367.78 kPa, which increased approximately 10 times with the increased amount of calcium carbonate + cement in the specimens. This strength is higher than the initial strength of the previous specimens.

The specimens produced by curing calcium carbonate + cement with 2% of the weight ratio for 3 days exhibited 1.4 times lower uniaxial compression strength than the strength of the untreated specimens. This is quite different from what we expected. It is thought that this resulted from the fact that a small amount of calcium carbonate and cement was not fully dried to inhibit cohesion to result in lowered strength. However, the increased weight ratio contributed to increasing the strength approximately 6.7–8.6 times with respect to the curing periods of 7 days, 14 days, 21 days, and 28 days. It was identified that the strength that increased approximately 1.3–3.0 times was attributable to longer curing period. The 2% and 4% calcium carbonate (low weight ratio) + cement specimens showed similar tendency to the increased strength of the untreated specimens with longer curing period. However, the 6% and 8% calcium carbonate (high weight ratio) + cement specimens showed higher strength than that of the cement specimens. The tendency showed that the calcium carbonate + cement specimens had a less effect of cement in the low weight ratio and in the early stage of curing but showed increased strength because of more cement contents in the specimens as the weight ratio increased. It is thought that this will contribute to a highly strong and environmentally safe admixture by studying the mixture of environment-friendly calcium carbonate produced through the reaction between cement and net microbes.

### 3.5. Results of Uniaxial Compression Test

The uniaxial compression strength of the calcium carbonate, cement, and calcium carbonate + cement specimens analyzed above was compared with respect to the different weight ratios (2%, 4%, 6%, and 8%). This was intended to compare and analyze the change of the uniaxial compression strength of calcium carbonate, cement, and calcium carbonate + cement specimens with respect to the same weight ratios. Figure 8 shows analysis and comparison for the effect of enhanced strength in the weak sandy soil ground by each cementation agent.

**Figure 11:**
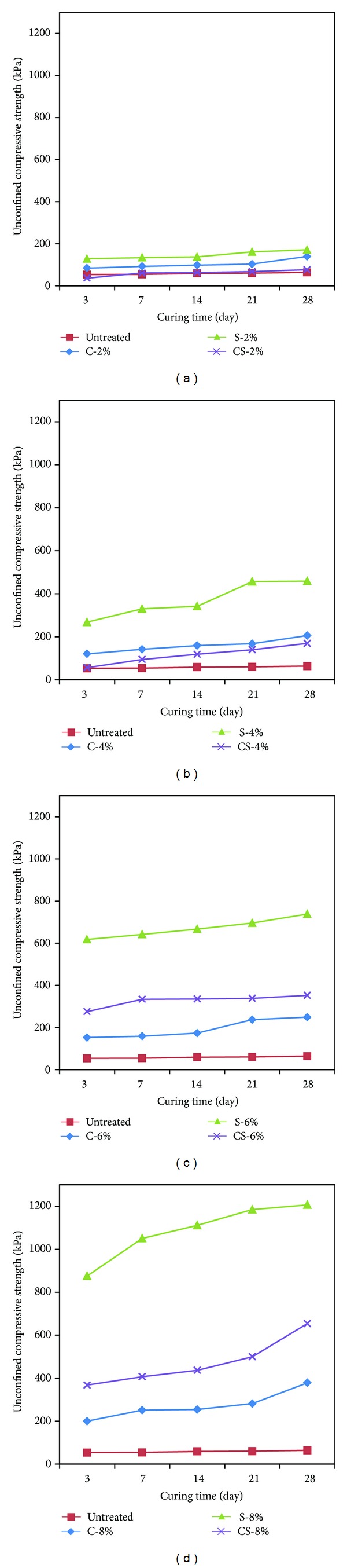
Strength of specimens by weight ratios.

Figures 11(a)–11(d) show the result of each specimen corresponding to (a) weight ratio 2%, (b) weight ratio 4%, (c) weight ratio 6%, and (d) weight ratio 8% for comparing the strength of each specimen depending on the weight ratio.

Figure 11(a) shows the result of weight ratio 2%. For weight ratio 2%, higher strength was measured in the order of untreated, calcium carbonate, cement, and calcium carbonate + cement specimens, but most specimens showed a similar tendency to the untreated specimens. It is thought that a small amount of the cementation agent does not significantly affect the enhancement of strength.

Figure 11(b) shows the result of weight ratio 4%. For weight ratio 4%, higher strength was measured in the order of untreated, calcium carbonate + cement, calcium carbonate, and cement specimens. The cement specimens showed 2.2–2.7 times higher strength than the calcium carbonate specimens. The calcium carbonate specimens showed 1.1–2.1 times higher strength than the calcium carbonate + cement specimens, but a similar tendency was shown. It is thought that because the cement contents of the calcium carbonate + cement specimens are smaller than those of the cement specimens, the calcium carbonate + cement specimens showed lower uniaxial compression strength than that of the calcium carbonate specimens.

Figure 11(c) shows the result of weight ratio 6%. For weight ratio 6%, higher strength of the specimens was measured in the order of untreated, calcium carbonate, calcium carbonate + cement, and cement specimens, unlike the uniaxial compression strength of the 2% and 4% specimens. The cement specimens showed 3.8–4.0 times higher uniaxial compression strength than that of the calcium carbonate specimens. The calcium carbonate + cement specimens showed 1.4–2.0 times higher uniaxial compression strength than that of the calcium carbonate specimens.

Figure 11(d) shows the result of weight ratio 8%. For weight ratio 8%, higher strength of the specimens was measured in the order of weight ratio 6%. The cement specimens showed 3.2–4.4 times higher uniaxial compression strength than that of the calcium carbonate specimens. The calcium carbonate + cement specimens showed 1.6–1.8 times higher uniaxial compression strength than that of the calcium carbonate specimens. The uniaxial compression strength of the highest weight ratio 8% specimens gradually increased as the amount of the cementation agent increased with the sand weight in a given specimen.

The analysis of the graph for the calcium carbonate and calcium carbonate + cement specimens reveals that the increase of the uniaxial compression strength with the increased weight ratio is not significant like the cement specimens. However, because longer period for curing the calcium carbonate and calcium carbonate + cement specimens tends to contribute to enhanced strength, it is thought that the effect of enhancing strength in the weak sand ground will be implemented.

### 3.6. Results of X-Ray Diffraction Analysis (XRD)

XRD analysis was carried out to identify ores created in the calcium carbonate produced by means of microbes, cement, and calcium carbonate + cement specimens. Each sample was reduced to fine powder, and the diffraction angle of X-rays was set as 5–70° for 2. For the calcium carbonate specimens, the result of identified silica (SiO_2_) and calcium carbonate (CaCO_3_) was described to compare the result of identification in the cement specimens. Silica (SiO_2_) which is a representative ore of cement and aluminum oxide (Al_2_O_3_) was identified in the cement specimens. The result of identification in the calcium carbonate + cement specimens includes silica (SiO_2_), aluminum oxide (Al_2_O_3_), and calcium carbonate (CaCO_3_).  Table 5 shows the result of XRD analysis for each specimen, which includes right minerals in each specimen. Note that the percentage of calcium carbonate is larger in the specimen with calcium carbonate than the specimen with calcium carbonate+cement.

## 4. Summary and Conclusions

This study aims to identify the effect of enhanced strength by calcium carbonate produced through microbial reaction in the sandy soil ground by means of the uniaxial compression test.

Specimens with weight ratios 2%, 4%, 6%, and 8% were produced to measure the uniaxial compression strength for the curing periods of 3 days, 7 days, 14 days, 21 days, and 28 days and to identify the type of minerals contained in the specimens by means of XRD analysis (Table 6).

1. The uniaxial compression strength was measured to be 84.57–378.86 kPa after curing the calcium carbonate specimens for each weight ratio for 3 days, 7 days, 14 days, 21 days, and 28 days. It was identified that the strength of calcium carbonate produced by means of microbial reaction increased approximately 1.5 times with the increased period of curing in comparison with initial curing. As the amount of calcium carbonate in specimens increased, their strength also increased. It is thought that shrinking for sand particles holding together due to water evaporation in the specimens contributed to increased uniaxial compression strength with the longer period of curing.
2. The strength of cement specimens was measured to be 128.45–1,206.69 kPa for each weight ratio after curing them for 3 days, 7 days, 14 days, 21 days, and 28 days. The analysis revealed that the reaction of hydration and the increased strength of the sandy soil were attributable to the reaction of hydration and Pozzolanic reaction as the amount of cement and the period of curing increased.
3. The uniaxial compression strength of calcium carbonate + cement specimens was measured to be 128.45–654.27 kPa for each weight ratio after curing them for 3 days, 7 days, 21 days, and 28 days. The mixture ratio of 1 : 1 of calcium carbonate produced by means of microbial reaction with cement resulted in less effect of cement in low weight ratio 2–4% specimens. However, it is thought that the enhanced strength of weight ratio 6~8% specimens is attributable to more cement contents in the specimens. It is expected that this will be an environment-friendly admixture to combine the environment-friendly calcium carbonate produced by means of net microbial reaction with cement.

It is thought that the strength of calcium carbonate, cement, and calcium carbonate + cement specimens increases for the sandy soil ground with the increased weight ratio and with longer period of curing them. For weight ratio 2%, calcium carbonate achieved better effect in terms of strength than calcium carbonate + cement. However, for weight ratio 4–6%, calcium carbonate + cement achieved more strength enhancement than calcium carbonate for the sandy soil ground. It is thought that this results from the fact that the increased weight ratio contributed to the increased amount of cement in the specimens to enhance strength by means of initial strength and the reaction of hydration. It is necessary to further study how to ideally mix calcium carbonate produced by means of microbes with cement to achieve higher strength of calcium carbonate + cement. Successful study will contribute to reducing the environmental costs for carbon dioxide emissions and excavation of lime.

## Figures and Tables

**Table 1:** Mixing the sample material.

Test ID	Cementation material	Soil (g)	Water (g)	Weight ratio (%)	Calcium carbonate (g)	Cement (g)	Quantity
Untreated	—	275	50	—	—	—	5

C-2%	Calcium carbonate	275	50	2	5.5	—	5
C-4%		275	50	4	11	—	5
C-6%		275	50	6	16.5	—	5
C-8%		275	50	8	22	—	5

S-2%	Cement	275	50	2	—	5.5	5
S-4%		275	50	4	—	11	5
S-6%		275	50	6	—	16.5	5
S-8%		275	50	8	—	22	5

CS-2%	Calcium carbonate + cement	275	50	2	2.25	2.25	5
CS-4%		275	50	4	5.5	5.5	5
CS-6%		275	50	6	11	11	5
CS-8%		275	50	8	16.5	16.5	5

**Table 2:** Curing period to compare strength of untreated specimens (unit: kPa).

Specimen	Curing period to compare strength of untreated specimens				
	3 days	7 days	14 days	21 days	28 days
Untreated	53.42	54.42	59.17	60.32	64.1

**Table 3:** Curing period to compare the strength of calcium carbonate specimens (unit: kPa).

Test ID	Curing period to compare the strength of calcium carbonate specimens				
	3 days	7 days	14 days	21 days	28 days
Untreated	53.42	54.42	59.17	60.32	64.10
C-2%	84.57	92.44	98.35	103.34	139.88
C-4%	120.69	142.10	159.60	168.16	205.78
C-6%	152.48	158.88	173.12	237.24	249.08
C-8%	199.95	251.43	254.20	281.71	378.86

**Table 4:** Curing period to compare the strength of cement specimens (unit: kPa).

Test ID	Curing period to compare the strength of cement specimens				
	3 days	7 days	14 days	21 days	28 days
Untreated	53.42	55.42	59.17	60.32	64.10
S-2%	128.45	134.17	137.58	161.41	170.99
S-4%	268.47	330.15	342.26	456.78	459.06
S-6%	618.13	641.79	666.99	695.76	738.54
S-8%	876.06	1050.73	1111.4	1184.69	1206.69

**Table 5:** Curing period to compare the strength of calcium carbonate + cement specimens (unit: kPa).

Test ID	Curing period to compare the strength of calcium carbonate + cement specimens				
	3 days	7 days	14 days	21 days	28 days
Untreated	53.42	55.42	59.17	60.32	64.10
CS-2%	36.80	61.12	62.79	67.83	76.45
CS-4%	56.93	94.78	118.81	140.19	169.56
CS-6%	275.26	333.96	335.70	338.75	352.78
CS-8%	367.78	407.14	436.60	499.94	654.27

**Table 6:** Specimen curing period in accordance with the results of XRD analysis.

Test ID	Formula	Scale factor	Score
C3-4%	SiO_2_	0.308	54
	CaCO_3_	0.124	12
C7-4%	SiO_2_	0.326	63
	CaCO_3_	0.286	34
C14-4%	SiO_2_	0.352	70
	CaCO_3_	0.354	12
C21-4%	SiO_2_	0.404	80
	CaCO_3_	0.450	10
C28-4%	SiO_2_	0.411	78
	CaCO_3_	0.556	12

S3-4%	SiO_2_	0.773	72
	Al_2_O_3_	0.097	22
S7-4%	SiO_2_	1.024	65
	Al_2_O_3_	0.271	21
S14-4%	SiO_2_	1.530	61
	Al_2_O_3_	0.278	11
S21-4%	SiO_2_	2.635	72
	Al_2_O_3_	0.301	17
S28-4%	SiO_2_	2.523	61
	Al_2_O_3_	0.329	7

CS3-4%	SiO_2_	0.962	73
	Al_2_O_3_	0.052	26
	CaCO_3_	0.152	11
CS7-4%	SiO_2_	0.995	79
	Al_2_O_3_	0.062	10
	CaCO_3_	0.152	16
CS14-4%	SiO_2_	0.999	67
	Al_2_O_3_	0.077	12
	CaCO_3_	0.162	10
CS21-4%	SiO_2_	1.010	50
	Al_2_O_3_	0.084	19
	CaCO_3_	0.172	8
CS28-4%	SiO_2_	1.100	72
	Al_2_O_3_	0.089	21
	CaCO_3_	0.175	11
